# Construction and characterization of thymidine auxotrophic (*ΔthyA*) recombinant *Lactobacillus casei* expressing bovine lactoferricin

**DOI:** 10.1186/s12917-018-1516-y

**Published:** 2018-06-27

**Authors:** Han Zhou, Xuechun Li, Zongying Wang, Jiyuan Yin, Hongchao Tan, Li Wang, Xinyuan Qiao, Yanping Jiang, Wen Cui, Min Liu, Yijing Li, Yigang Xu, Lijie Tang

**Affiliations:** 1 College of Veterinary Medicine, Northeast Agricultural University, Chang Jiang road No. 600, Xiang Fang District, Harbin, China; 2 Heilongjiang Key Laboratory for Animal Disease Control and Pharmaceutical Development, Harbin, China; 3 College of Animal Science Technology, Northeast Agricultural University, Harbin, China

**Keywords:** Recombinant *Lactobacillus casei*, Thymidine auxotrophy, Expression of Lfcin

## Abstract

**Background:**

*Lactobacillus casei* (*L. casei*) is well known for its probiotic property in human and animals. Lactoferricin (Lfcin) polypeptide can effectively modulate host immune responses and have antimicrobial activity in vivo and in vitro. In order to develop a food-grade *L. casei* system constitutively expressing bovine Lfcin, this study constructed a thymidine auxotrophy (*ΔthyA*) recombinant *L. casei*.

**Results:**

Based on the thymidylate synthase gene (thyA) insert site, LFEC(Lfcin expression cassette)was inserted into *L. casei* genome through homologous recombination, successfully expressed and could be stably inherited. The recombinant *L. casei*, *ΔthyA L. casei*-LFEC, is sensitive to chloramphenicol and limited when cultured without thymine. Meanwhile, *ΔthyA L. casei*-LFEC has both good antibacterial activity against *Escherichia coli* and *Staphylococcus aureus* and antiviral activity against porcine epidemic diarrhea virus (PEDV).

**Conclusions:**

We successfully constructed a recombinant *L. casei* strain expressing Lfcin, *ΔthyA L. casei*-LFEC, which could only survive in the presence of thymine, and had excellent antimicrobial and antiviral activity with good genetic stability and sensitivity. This research provides a cost-effective alternative to the antibiotics with additional biological functions and wider applicability prospect. Using *ΔthyA* as the selectable mark instead of antibiotic to construct genetic engineering *L*.*casei* provides a safe and effective approach of feed additives in livestock raising.

## Background

*Lactobacillus casei* (*L. casei*) is well known as a kind of gram positive bacteria with its probiotic property for human and animals, which can maintain microflora homeostasis, inhibit pathogens growing and regulate pH balance in the host gastrointestinal environment [1]. Compared to *Escherichia coli* (*E. coli*) expression system, the most significant advantage of *L. casei* expression system is that the genetic engineered *L. casei* with vaccine and pharmaceutics purposes can be directly applied via oral administration [2–4]. However, antibiotic resistances are common used as selectable mark for the construction of genetic engineering *L. casei* expression systems [5], which would result in potential risk to the environment and human.

Thymidylate synthase encoded by thymidylate synthase gene (*thyA*) is a kind of highly conserved isozyme present in different bacteria, which plays a crucial role in DNA synthesis, and works as the key enzyme in de novo synthesis of phosphorylated deoxythymidine uracil (dTMP) by catalyzing deoxyuridine ribonucleotides (dUMP) into dTMP via methylation modification [5, 6]. When the *thyA* is deleted, the DNA de novo synthesis pathway in *L. casei* will be blocked, resulting in proliferation failure [7]. Meanwhile, addition with thymidine or thymine in culture as the substrate for dTMP synthesis may promote the growth of *L. casei* [5].

Lactoferricin (Lfcin) polypeptide is dissociated off from lactoferrin under acidic condition, which can effectively modulate host immune responses, such as recruiting and promoting the balance of the production of immune cells [8]. The Lfcin has antimicrobial activity in vivo and in vitro [9]. Lfcin with positive charges could establish nonspecific binding with the lipid layer carrying negative charges in cell walls, and then induce autolysis death of bacteria cell by increasing its membrane permeability [10]. Moreover, the Lfcin could block the iron intake of microorganisms to act antimicrobial activity [11]. The Lfcin also possesses many other probiotic properties, such as antioxidation, antiviral activity, inhibiting tumor cell growth and regulating the immunity of the organism [12–16].

In this study, we successfully constructed a genetic engineering *L*.*casei* using *ΔthyA* as the selectable mark instead of antibiotic, followed by the expression of bovine Lfcin as a multifunctional protein, suggesting a safe and effective approach for feed additives of livestock or in other industries.

## Methods

### Bacterial strains, cell strain and virus strain

Bacterial strains used in this study are listed in Table 1. *L. casei* and *ΔthyA L. casei* was cultured in GM17 broth supplemented with 40 μM of thymidine at 37°C. The temperature-sensitive plasmid pGBHCupp was constructed in our laboratory, containing a pWV01 replicon and chloramphenicol resistance genes. VERO cell line (ATCC® CCL-81™, USA) was stored in our laboratory. PEDV LJB/03 was preserved in our laboratory at − 80°C.

**Table 1:** Bacterial strains and plasmids used in this study

	Genotype / characteristics
*E. coli* strains	
TG1	*E. coli* cloning host
pMD18-T-HCE-MCS-rrnBT1T2/TG1	TG1harbring pMD18-T-HCE-MCS-rrnBT1T2,Cmr^r^
pGBHC-Pupp/TG1	TG1 harboring pGBHC-Pupp, Cmr^r^
pUC57-*LFcin*/JM109	JM109 harboring pUC57-*Lfcin,* Am^r^ (Synthesis by Genewiz Biological Technology Co., Ltd)
pGBHCupp-TF-LFEC-TR/TG1	TG1 harboring pGBHCupp-TF-ELFEC-TR, Cmr^r^
*L. casei*strains	
*ΔuppL.casei*ATCC393	*L. casei* ATCC393 derivative without *upp*
pGBHCupp-TF-LFEC-TR/*L.casei*	*ΔuppL.casei*ATCC393 harboring pGBHCupp-TF-ELFEC-TR, Cmr^r^
*ΔthyAL.casei-*LFEC	Δ*thyAL.casei* ATCC393 with *LfcinB*expression cassette insertion in the *thyA*
Plasmids	
pGBHC-upp	Ori (pWV01) with a copy of upp expression cassette, Cmr^r^
pGBHCupp-TF-LFEC-TR	pGBHC-upp containing *LfcinB* expression cassette between upstream and downstream sequence flanking the *thyA* integration site

### Construction of the pGBHCupp-TF-LFEC-TR

**Fig. 1:**
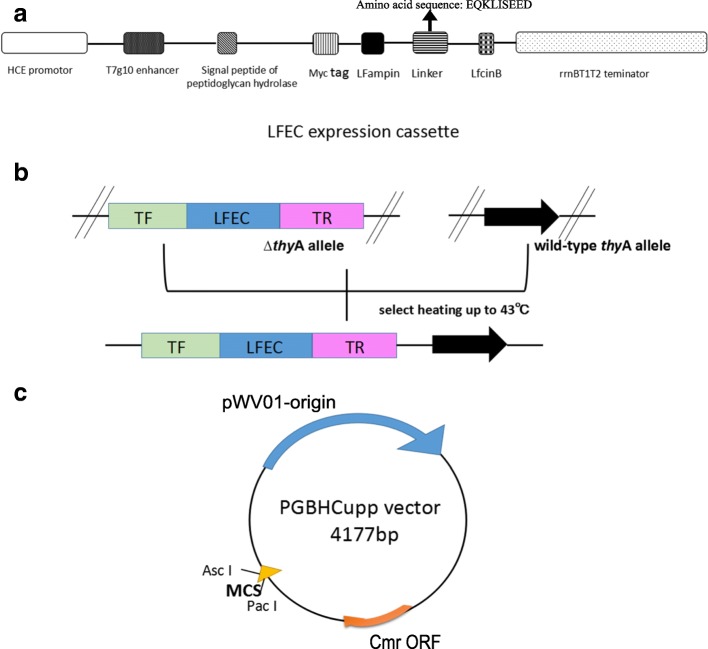
Construction diagram of integration plasmids pGBHC-upp-LFEC. **a** Schematic diagram of LFEC. **b** Schematic diagram of the insertion of LFEC between homologous arms. The wild-type thyA was replaced by LFEC through homologous recombination, which occurs after the temperature reaches 43 °C due to the application of the temperature-sensitive plasmid. **c** Schematic diagram of the insertion of LFEC into pGBHCupp plasmids

The HCE promoter, T7 g10 enhancer, signal peptide of peptidoglycanhydrolase and Myc tag, and the two segments of bovine Lfcin (LFcinB and Lfampin) connected with linker were synthesized and inserted into pUC57 vector by Genewiz Biological Technology Company, Ltd., Beijing, China. The recombinant plasmid was named pUC57–Lfcin. The terminator rrnBT1T2 gene was amplified by PCR using pMD18-T-HCE-MCS-rrnBT1T2 plasmid DNA as template. The rrnBT1T2 fragment was gel purified, inserted into pMD19-T-vector (Takara, DaLian, China), and named pMD19-TS-rrnBT1T2. To construct LFEC(Lfcin expression cassette), Lfcin was digested off from pUC57–Lfcin and inserted into pMD19-TS-rrnBT1T2 (Fig. 1a). The recombinant plasmid was named pMD19-TS-LFEC.

For homologous recombination in *L. casei* 393, upstream homologous arm TF and downstream homologous arm TR were PCR amplified using *L. casei* 393 genomic DNA as a template to create an internal deletion in the *thyA* site. The two fragments were gel purified, inserted into the pMD19-T vector and named pMD19-TS-TFTR. Then, the LFEC was digested off from pMD19-TS-LFEC, inserted into pMD19-TS-TFTR and named pMD19-TS-TF-LFEC-TR.

The TF-LFEC-TR was digested off from pMD19-TS-TF-LFEC-TR, inserted into pGBHCupp vector (Fig. 1c) and named pGBHCupp-TF-LFEC-TR, which was both temperature sensitive and chloramphenicol resistant.

PCR primers used for amplifying target genes are listed in Table 2.

**Table 2:** Primers used in this study

Primer	Primer sequence (5′-3′)	Product size
Cloning primers		
rrnBT1T2	F:ACTAGTGTCAATGATGAGATCTGGCTGTTTTGGCGGATGAGAGA R:CTCGAGAGAGTTTGTAGAAACGCAAAAAGGC	438 bp
LFEC	F:TCTAGAGATCTCTCCTTCACAGATTCCCAAT R:CTCGAGAGAGTTTGTAGAAACGCAAAAAGGC	1022 bp
TF	F: GGCGCGCCTTAGGCGAGACCGTTCAT R:TTAATTAACGTATGACGCACTAGTCGATCAGGCTTGAAATGG	1020 bp
TR	F:ACTAGTGACTAGCGATCTCGAGCACGCATACAGGCACGTA R: TTAATTAAGGGTCACGAGCAAGGTAT	1392 bp
Screening primers		
*thyA*	F: TGTGGCATCACTTAGGACC R: TGTGGCATCACTTAGGACC	3268 bp
Real-time PCR primes		
PEDV-N	F:ACTGAGGGTGTTTTCTGGGTTGC R:GGTTCAACAATCTCAACTACACTGG	137 bp
Beta-actin	F:AAGGATTCATATGTGGGCGATG R:TCTCCATGTCGTCCCAGTTGGT	103 bp

Enzyme restriction sites are underlined

### Electrotransformation

Briefly, 100 ml culture of *L. casei* cells was grown in MRS medium at 37°C for 4 h. The cells were ice-bathed for 10 min, harvested by centrifugation at 5000 g for 10 min at 4°C, and resuspended in 20 ml of ice-cold EPWB buffer. Then, the cells were harvested and resuspended in ice-cold EPWB buffer for two more times as described above. Finally, the competent cells were harvested and resuspended in 1 ml EPB buffer. 200 ng of pGBHCupp-TF-LFEC-TR plasmid was added to 200 μl of competent cells in ice bath and transformed by electroporation at 2.0 kV. Cells were recovered in MRS medium for 2–3 h and then spread on MRS agar plate containing 2.5 μg/ml of chloramphenicol. The positive colony was identified and the recombinant pGBHCupp-TF-LFEC-TR/*L.casei* was obtained.

### Homologous recombination of *L*. casei ATCC 393

The process of homologous recombination was followed with temperature sensitive selection (Fig. 1b). The integration transformants were transferred (1% inoculum) three times at 43°C and grown to stationary phase each time to select single-crossover integrations, after which the pGBHCupp vector containing chloramphenicol resistant gene stopped replication. Then, the single-crossover integrations (1% inoculums) were propagated in GM17 broth for 30 generations in the absence of antibiotic resistance to achieve the second homologous crossover and lose the recombinant plasmid. The presence of the LFEC insertion in *ΔthyA L. casei* chromosome was detected by PCR with the primers *thyA* F and *thyA* R shown in Table 2. The positive integration *L. casei* was named as *ΔthyA L. casei*-LFEC.

### Test of auxotrophy and chloramphenicol-sensitivity

In order to test the dependence of *ΔthyA L. casei*-LFEC on thymine, the *ΔthyA L.casei*-LFEC was cultured overnight in GM17 broth supplemented with thymineand spread on GM17 agar plates in the presence or absence of thymine for incubation at 37°C for 48 h. Then, the bacteria concentration was determined. In parallel, *L.casei* was used as control. In order to detect plasmids residual in *ΔthyA L.casei*-LFEC for chloramphenicol resistance, the bacteria cultured overnight in MRS broth supplemented with thymine was spread on MRS agar plates in the presence or absence of chloramphenicol at 37°C for 48 h to observe.

### Growth kinetics of the *ΔthyA L.casei*-LFEC

In order to analyze growth kinetics of the *ΔthyA L.casei*-LFEC, the growth curve of the *ΔthyA L.casei*-LFEC was determined in the presence or absence of thymine. The bacteria density was measured by absorbance at OD_600_ every 2 h until 48 h. In parallel, *L. casei* cultured in GM17 broth in the presence or absence of thymine was used as control.

### Stability of the *ΔthyA L.casei*-LFEC

To determine the recombinant strain stability post chromosomal integration, the culture of *ΔthyA L.casei*-LFEC (1% inoculum) was continuously transferred 50 generations at interval of 12 h, then genomic DNA of each generation was extracted and detected by PCR for the presence of LFEC. PCR primers used in this study are listed in Table 2.

### Determination of Lfcin expressed by ΔthyA L.casei-LFEC

Western blot assay was performed to analyze the expression of Lfcin in *ΔthyA L.casei*-LFEC cultured in GM17 broth supplemented with thymine. Following the extraction of total proteins in 12% sodium dodecyl sulfate-polyacrylamide gel electrophoresis (SDS-PAGE), the proteins were electro-transferred onto a nitrocellulose membrane, incubated with mouse anti-LfcinB antibody prepared in our lab and horseradish peroxidase (HRP)-conjugated goat anti-mouse IgG antibody diluted at 1:5000 (Invitrogen, USA), and visualized with a chemiluminescent substrate reagent according to the manufacturer’s instruction [17, 18].

Indirect immunofluorescence assay was performed to detect the expression of Lfcin on the cell surface of *ΔthyA L.casei*-LFEC as previously described [19]. Briefly, 1 mL of *ΔthyA L.casei*-LFEC cultured in GM17 broth for 12 h was centrifuged, washed with PBS three times, and resuspended in 1 mL of sterile PBS-3% bovine serum albumin (BSA) containing mouse anti-myc antibody; following the incubation at 37°C for 1 h, the cells were harvested, washed three times, and incubated in 1 mL of FITC-conjugated goat anti-mouse IgG antibody (diluted at 1:500) at 37°C for 1 h; then, the cells were washed three times, transferred onto a glass slide, and fixed with cold acetone for 30 min; Confocal microscope was used to observe fluorescence signals. In parallel, *L. casei* was used as negative control.

The Lfcin concentration in the supernatant of *ΔthyA L.casei*-LFEC was determined using the native bovine Lfcin (XingHao Pharmaceutical co., ltd., WuHan, China) as standard sample and anti- Lfcin monoclonal antibody (preserved in our lab) as detection antibody.

### Antimicrobial activity test of Lfcin

To determin the antimicrobial activity of Lfcin, single bacterial colony of *E. coli* and *S. aureus* were inoculated in LB broth respectively, then 10 mL of filtered (0.22 μm) supernatant of *ΔthyA L.casei*-LFEC was added when the OD_600_ value reached 0.3–0.4. In comparison, the supernatant of *L.casei* culture was used as negative control. Bacteria concentration of each group was detected by determining OD_600_ value at intervals of 2 h. At every time point, the colony counting of *E. coli* and *S. aureus* were performed and the inhibition percentages were calculated.

### Transmission electron microscopy (TEM)

To study the insight of the direct effects of Lfcin in the morphology of bacterial cells, logarithmic growth phase of *S. aureus* and *E. coli* cells after treatment with the supernatant of *ΔthyA L.casei*-LFEC were chosen to assess the bacterial membrane damage by TEM. After incubation, the cells were pelleted by centrifugation at 1000 rpm for 5 min, followed by washing thrice with PBS. Subsequently, the cells were fixed with 2.5% glutaraldehyde for 1 h, washed in PBS for three times, centrifuged in an series of increasing ethanol (30, 50, 70, 90% and absolute ethanol) 20–25 min [20]. The cells were penetrated with acetone and embedding agent at a 1:1 volume, shaken for 2 h by an oscillator (Qilinbeier, Jiangsu, China), and again shaken for 2 h in the pure embedding agent before polymerization in the incubator at 37°C for 24 h, 45°C for 48 h, and 60°C for 48 h. Next, 120 nm ultra-thin slices were sectioned and stained with 4% uranyl acetate for 20 min and with double electron staining with lead citrate for 5 min. These ultra-thin sections were then placed on a single-hole copper mesh and were subjected to observation and photography under electron microscopy.

### Antiviral activity test of Lfcin

VERO cells were grown in 96-wells tissue culture plates at 37°C in 5% CO_2_ until 85% confluence and infected with PEDV at 1.0 MOI for 1 h at 37°C [21]. For the experiment groups, 100 μL filtered supernatant of Δ*thyA L.casei*-LFEC was added to VERO cells before PEDV absorption, simultaneously with PEDV and after PEDV absorption. For the control groups, 5.0 ng/L of native bovine Lfcin was added as positive control, while the filtered supernatant of *L.casei* was added as negative control. VERO cells were frozen and thawed for three times at 72 hpi, after which total RNA was extracted using Fast2000 RNA kit and cDNA was gained using Reverse Transcription enzyme (Toyobo, Japan). Real-time fluorescent quantitative PCR was performed to detect the viral replication using FastStart Universal SYBR Green Master (Roche, Switzerland) and ABI 7500 real time PCR system was used to determine the viral replication.

### Statistical analysis

The results were analyzed as the (1/2^-*ΔΔCt*^) ± SD [21], the*ΔΔCt* = (Ct_PEDV N gene-_Ct_β-actin_) _Lfcin_/(Ct_PEDV N gene-_Ct_β-actin_)_negative control_. Comparisons between groups were performed using analysis of Tukey’s. The *P* value of < 0.05 (*P* < 0.05) was considered as statistically significant and *P* < 0.01 as highly significant.

## Results

### Construction of the *ΔthyA L.casei*-LFEC

**Fig. 2:**
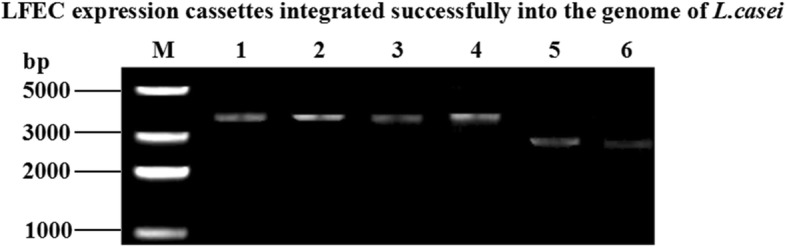
Identificaiton of the insertion of LFEC into *Δthy*A *L. casei* genome. M: DNA marker; Lanes 1–4: PCR results for *Δthy*A *L.casei* with LFEC insertion; Lanes 5–6: PCR results for *Δthy*A *L. casei* without LFEC insertion

LFEC was inserted between the homologous arms (TF and TR) as indicated in Fig. 1b. As confirmed by PCR results (Fig. 2), following the construction of pGBHC-upp-TF-LFEC-TR/*L.casei*, the LFEC constitutively expressing Lfcin was successfully inserted into *L.casei* genome via homologous recombination.

### Test of auxotrophy and chloramphenicol-sensitivity of *ΔthyA L.casei*-LFEC

**Fig. 3:**
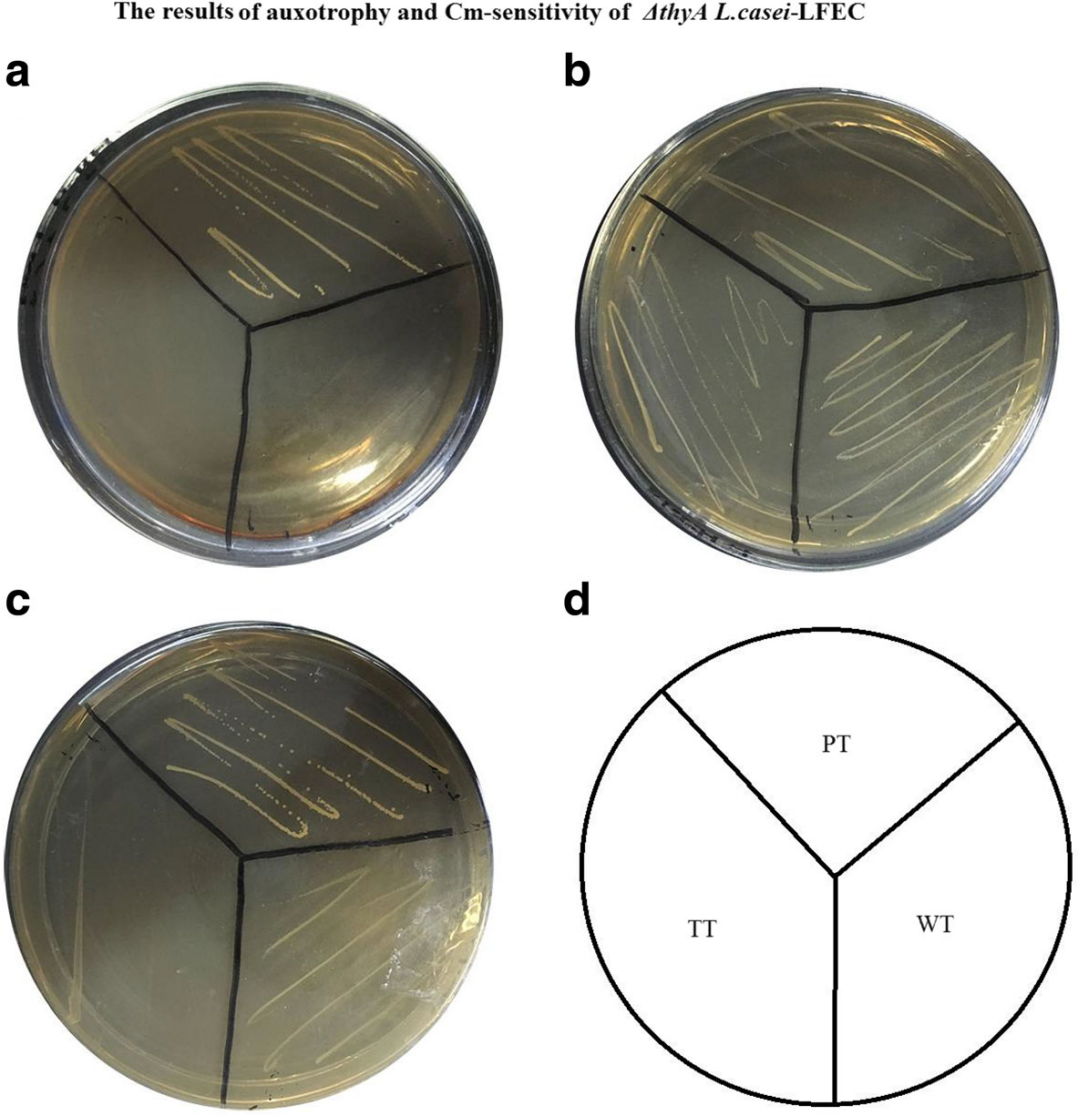
Detection of thymidine auxotrophy and chloramphenicol-sensitivity of *Δthy*A *L.casei*-LFEC. **a** GM17 plate containing both chloramphenicol and thymine; (**b**) GM17 plate containing thymine but no chloramphenicol; (**c**) GM17 plate containing neither chloramphenicol nor thymine; (**d**) Diagram of different strains: WT, *L.casei*; PT, pGBHCupp-LFEC/*L.casei*; TT, *ΔthyA L.casei*-LFEC

The results showed that only pGBHCupp-TF-LFEC-TR/*L.casei* (PT) could grow on the GM17 plate supplemented with Cmr and thymine (Fig. 3a), indicating no residual antibiotic resistance present in *ΔthyA L.casei*-LFEC; on the GM17 plate supplemented with thymine and without Cmr, *L. casei*, pGBHCupp-TF-LFEC-TR /*L.casei* and *ΔthyA L.casei*-LFEC could normally grow (Fig. 3b); on the GM17 plate without thymine and Cmr, *L. casei* and pGBHCupp-TF-LFEC-TR/*L.casei* could normally grow, but not *ΔthyA L.casei*-LFEC (Fig. 3c). Our results indicated that the *ΔthyA L.casei*-LFEC without Cmr resistance was constructed successfully.

### Determination of growth kinetics of *ΔthyA L.casei*-LFEC

**Fig. 4:**
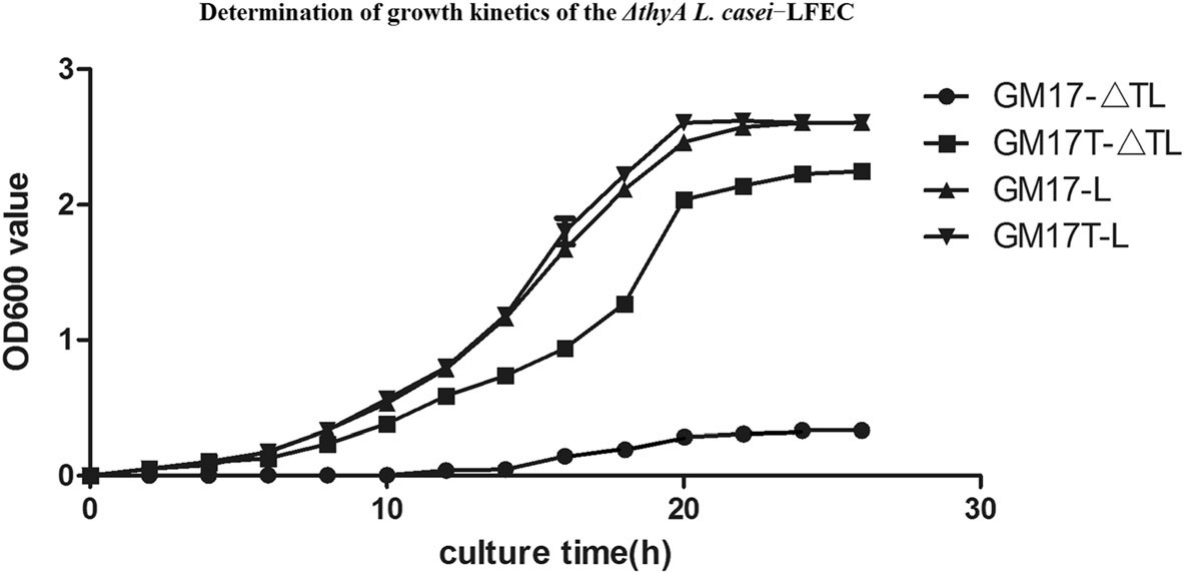
Growth kinetics of *ΔthyA L.casei*-LFEC in different GM17 medium using *L. casei* as control. GM17-ΔTL, growth curve of *ΔthyA L. casei*-LFEC in GM17 without thymine; GM17T-ΔTL, growth curve of *ΔthyA L. casei*-LFEC in GM17 with thymine; GM17-L, growth curve of *L. casei* in GM17 without thymine; GM17T-L, growth curve of *L. casei* in GM17 with thymine

Compared to wild type *L. casei*, *ΔthyA L.casei*-LFEC could grow normally with the addition of thymine in GM17 broth. However, the growth of *ΔthyA L.casei*-LFEC was limited in the absence of thymine, indicating the thymine dependence of *ΔthyA L.casei*-LFEC growth (Fig. 4).

### Genetic stability of *ΔthyA L.casei*-LFEC

**Fig. 5:**
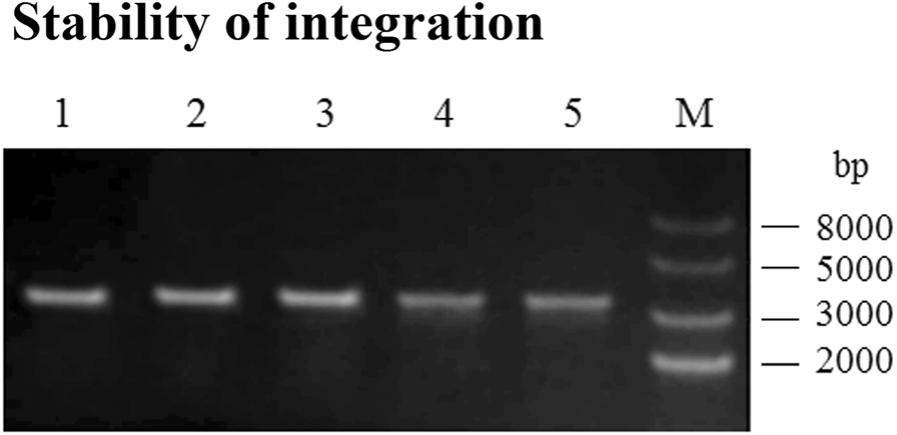
Detection of **t**he genetic stability of *ΔthyA L. casei*-LFEC. M: DNA marker; Lanes 1–5: PCR amplification results of LFEC of the 10 th to the 50 th generation of *ΔthyA L. casei*-LFEC

The *ΔthyA L.casei*-LFEC was serially cultured for 50 generations and the presence of the LFEC integrant was detected by PCR. As shown in Fig. 5, the LFEC was still detectable in the *thyA* location of *Δthy*A *L.casei*-LFEC, indicating good genetic stability.

### Expression of Lfcin by *ΔthyA L.casei*-LFEC

**Fig. 6:**
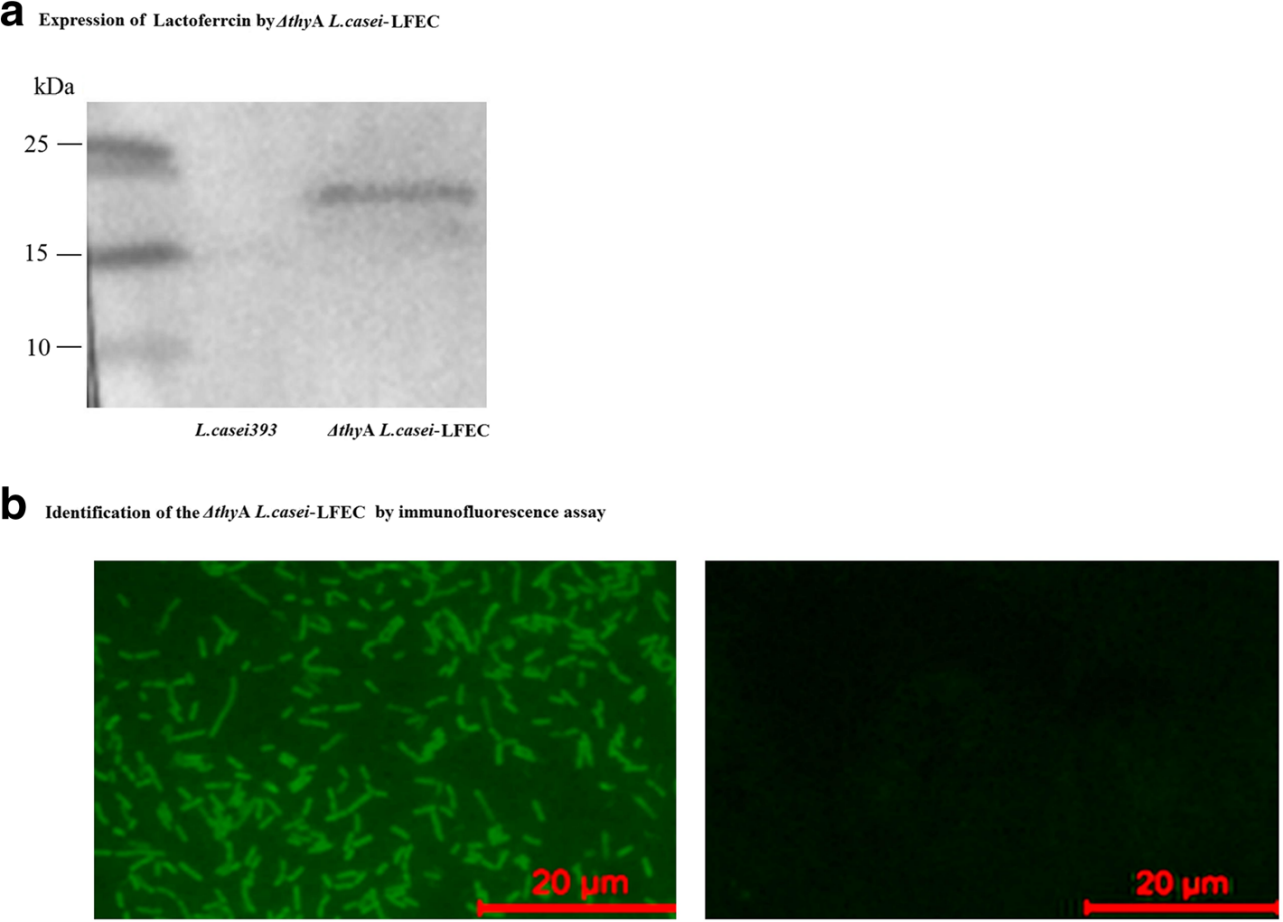
Expression identification of LFcin of *ΔthyA L. casei*-LFEC. **a** Western blot identification: Left lane, marker; Middle lane, *L. casei*; Right lane, *ΔthyA L. casei*-LFEC; (**b**) Indirect immunofluorescence identification: Left, *ΔthyA L. casei*-LFEC; Right, *L. casei*

Western blot analysis of Lfcin expression showed an immunoblot band of expected size (Fig. 6a), while indirect immunofluorescence results showed obvious fluorescence on the cell surface of *Δthy*A *L.casei*-LFEC (Fig. 6b). Both experiments confirmed the successful expression of Lfcin in *Δthy*A*L.casei*-LFEC. The Lfcin concentration in the supernatant of *ΔthyA L.casei*-LFEC was determined to be 23.37 μg/mL.

### Antibacterial activity of Lfcin expressed by *ΔthyA L.casei*-LFEC

**Fig. 7:**
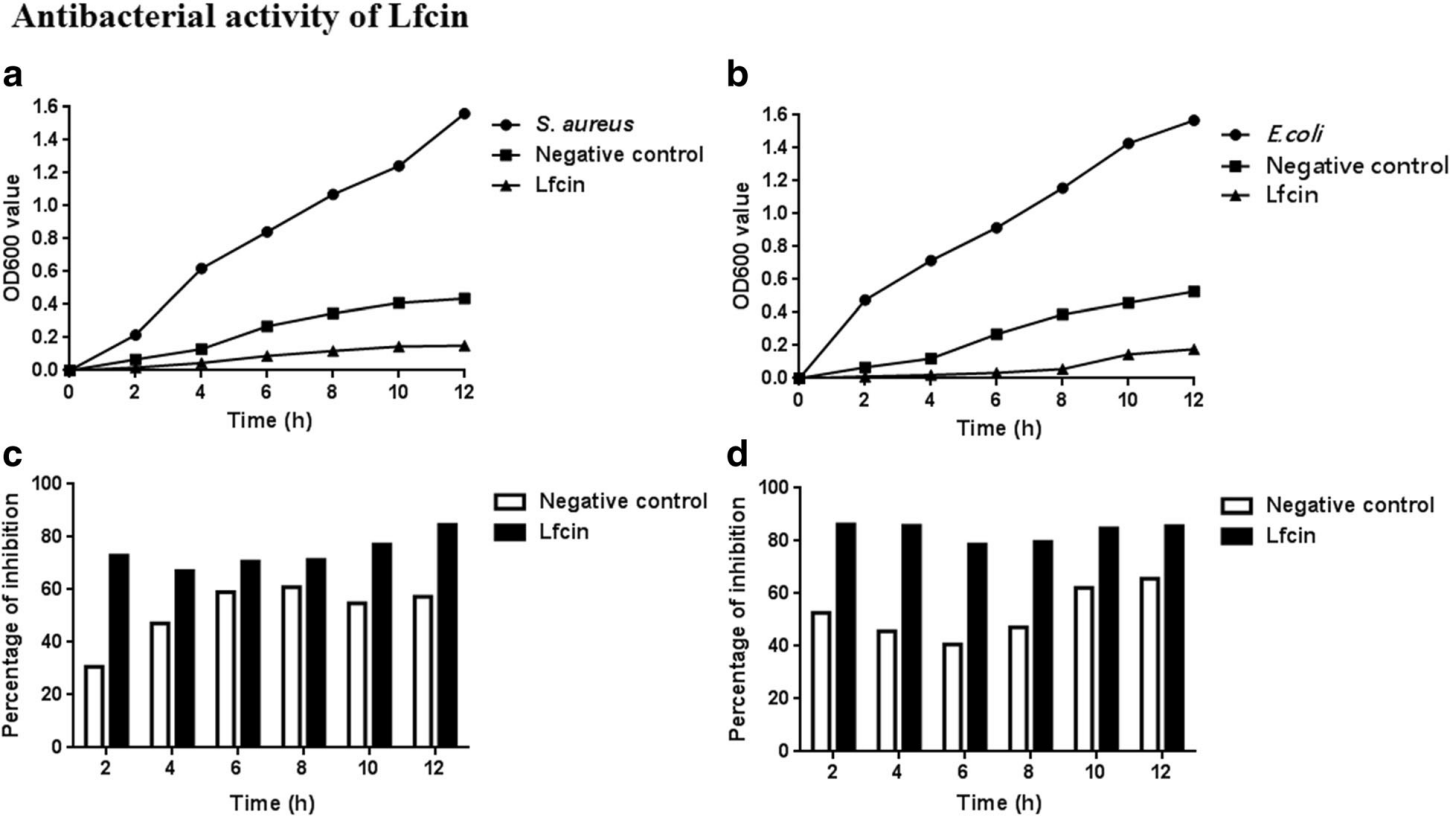
Antibacterial activity analysis of Lfcin. **a** *S. aureus*, growth curve of *S. aureus*; Negative control, growth curve of *S. aureus* cultured with *L. casei* filtered supernatant; Lfcin, growth curve of *S. aureus* cultured with *ΔthyA L. casei*-LFEC filtered supernatant; (**b**) *E. coli*, growth curve of *E. coli*; Negative control, growth curve of *E. coli* cultured with *L. casei* filtered supernatant; Lfcin, growth curve of *E. coli* cultured with *ΔthyA L. casei*-LFEC filtered supernatant; (**c**) Inhibition percentage of *S. aureus* cultured with *ΔthyA L. casei*-LFEC filtered supernatant; (**d**) Inhibition percentage of *E. coli* cultured with *ΔthyA L. casei*-LFEC filtered supernatant

**Fig. 8:**
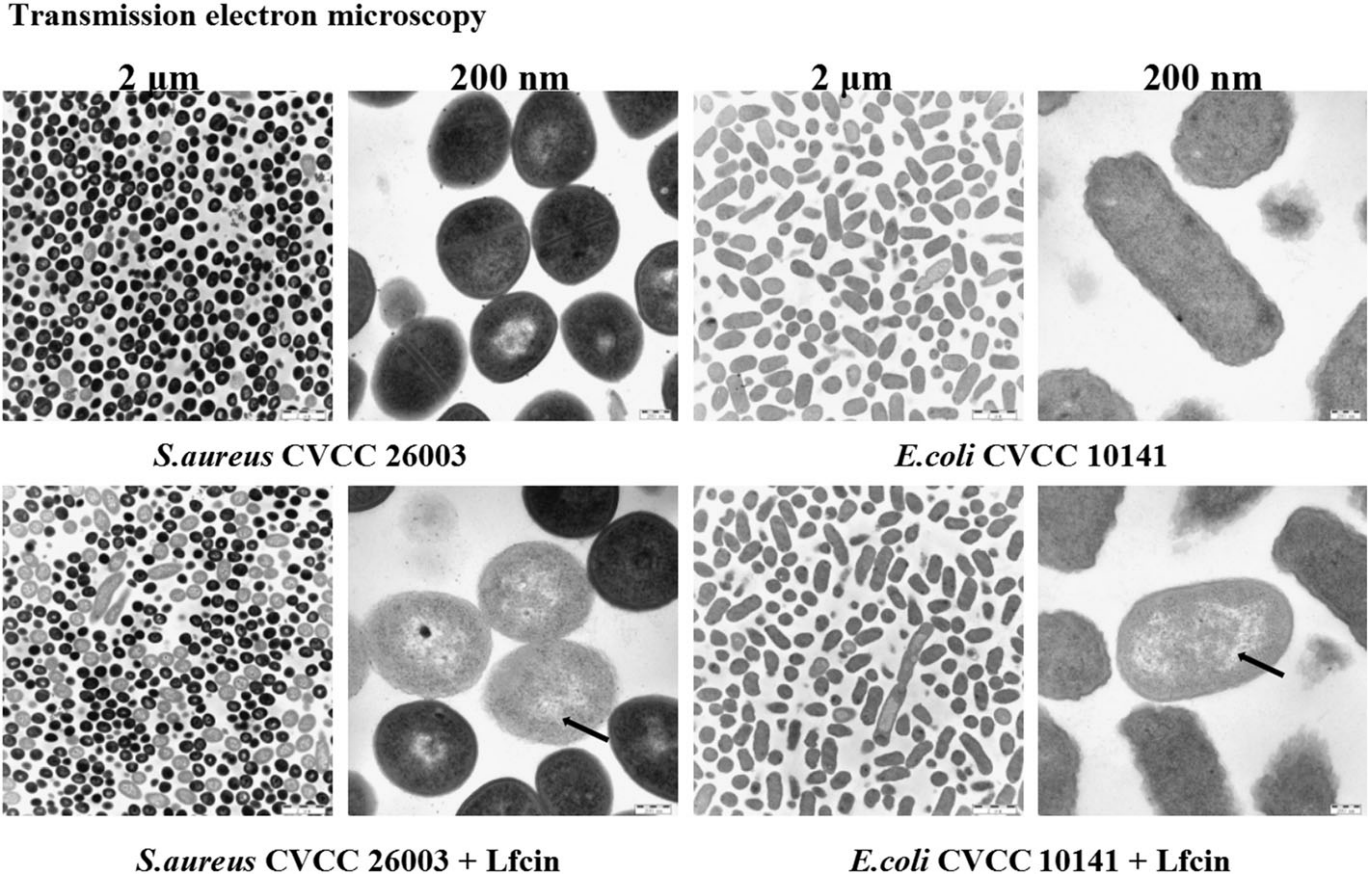
Transmission electron microscopy display of the antibacterial activity of Lfcin. **a, c** Untreated *S. aureus*; (**b, d**) *S. aureus* treated with Lfcin. (**e, g**) Untreated *E. coli*; (**f, h**) *E. coli* treated with Lfcin

Compared to wild type *L. casei*, the growth of both *S. aureus* and *E. coli* were obviously limited and the inhibition percentage were 42.22 and 40.05% higher respectively when cultured with Lfcin, indicating a strong antibacterial activity of Lfcin (Fig. 7). Meanwhile, TEM results showed that Lfcin lead to damages in cell morphology of *S. aureus* and *E. coli*, including rough cell surface and cell lysis (Fig. 8b, d, f and h), which indicated the potential bactericidal effect of Lfcin.

### Antiviral activity of Lfcin expressed by *ΔthyA L.casei*-LFEC

**Fig. 9:**
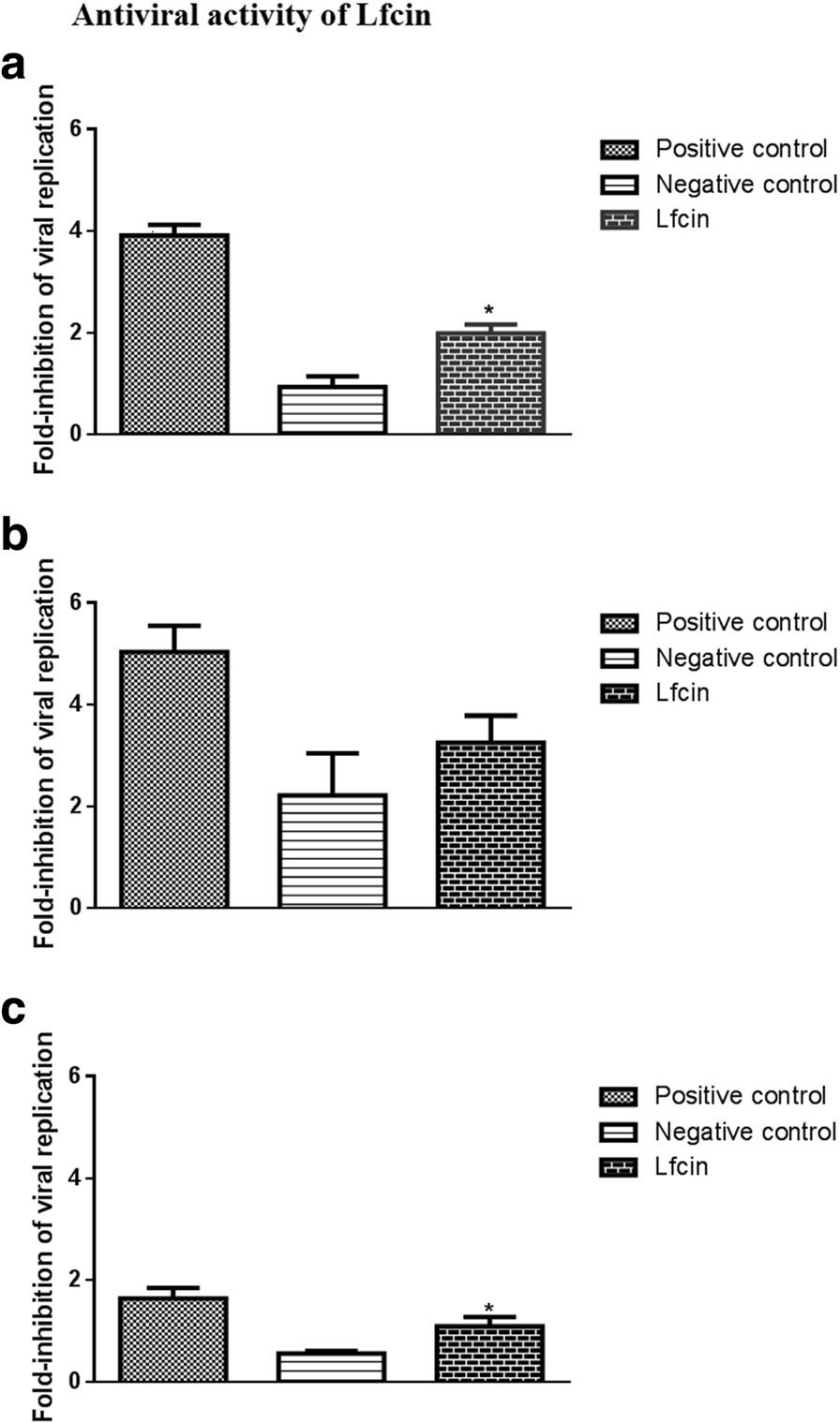
Antiviral activity analysis of Lfcin expressed by *ΔthyA L. casei*-LFEC. Positive, the inhibition of PEDV replication with the addition of native bovine lactoferricin; Negative control, the inhibition of PEDV replication with the addition of the supernatant of cultured *L. casei*; Lfcin, the inhibition of PEDV replication with the addition of the supernatant of cultured *ΔthyA L. casei*-LFEC; (**a**) The supernatant was incubated with VERO cells before PEDV adsorption; (**b**) The supernatant were simultaneously added to VERO cells with PEDV; (**c**) The supernatant were added to VERO cells after PEDV adsorption

The effect of Lfcin on the cellular receptors and viral proteins was tested with simultaneous incubation conditions. A significant inhibition of viral replication was observed when native bovine Lfcin or Lfcin expressed by *Δthy*A *L.casei*-LFEC were added with viral together, compared to the negative control (Fig. 9).

## Discussion

Using genetic engineered lactic acid bacteria (LAB) to deliver functional protein is a promising approach especially for oral administration development, which could effectively protect protein from protease digestion and induce effective functions [22–25]. LAB expression system has been widely used in oral vaccine development, whose effect has been proven both in vitro and in vivo. Anbazhagan et al. generated a recombinant LAB that constitutively overexpressed *B. subtilis* oxalate decarboxylase and degraded oxalate efficiently under in vitro conditions [26]. Moreover, Giselli et al. constructed a recombinant LAB that could secrete attenuated recombinant staphylococcal enterotoxin B and induce a protective immune response in a murine model of *S. aureus* infection [27]. Currently, for the construction of LAB expression system, some antibiotic resistances are widely used as selection marker. However, with the application of genetic engineered LAB harboring, the antibiotic resistance genes would be exposed to the environment and animals, causing potential risk to human.

In this study, we successfully constructed a *thyA*-based auxotrophy genetic engineered *L. casei* (*ΔthyA L. casei-*LFEC) with the deletion of antibiotic resistance gene as selection marker. The growth of *thyA* auxotrophy *L. casei* is high limited by the additional adding thymine or thymidine in the culture media, showing high thymine dependence. Compared to the antibiotic resistance selection marker, the most significant advantage of auxotroph selection marker is the safety to the environments. Thus, the *ΔthyA L. casei-*LFEC constructed in this study would provide a promising approach for food-grade agents’ development.

Lfcin is a kind of polypeptide with diverse biological functions, such as antibacterial effect, antiviral activity, antitumor effect and immunity modulation, suggesting a potential agent for food additive or immunologic adjuvant [28]. Bovine Lfcin was usually produced in vitro by yeast or *E. coli* [29, 30], while the complex purification process is required. Using lactic acid bacteriato deliver it suggested an alternative approach for utilizing Lfcin via oral administration. In the present study, by using the *ΔthyA L. casei* as delivery carrier and the Lfcin as the target functional protein, a genetic engineered *L. casei* constitutively expressing Lfcin, *ΔthyA L. casei-*LFEC, was constructed through two-step homologous recombination. The recombinant *ΔthyA L. casei-*LFEC was passed for 50 generations and showed good genetic stability. In our previous study, wild-type *L. casei* has already been applied to construct chromosomal insertion strains using homologous recombination which showed similar genetic stability to this study [25].

Furthermore, the antimicrobial activity and the antiviral activity of the Lfcin were evaluated in vitro. The growth curve results of *E. coli* and *S. aureus* indicated that the growth of both pathogenic bacteria were significantly inhibited when cultured with Lfcin*.* Also, *E. coli* and *S. aureus* showed cell damages in morphology in TEM experiment, which confirmed the antibacterial activity of Lfcin expressed in *ΔthyA L. casei-*LFEC. Meanwhile, TEM results showed that Lfcin caused changes in cell membrane permeability which lead to nucleic acid area leak and the loss of bacteria pathogenesis. In addition, the antiviral effect of Lfcin on PEDV replication was evaluated by determining the inhibition fold-chage of PEDV replication in three different infection phases treated with Lfcin. Lfcin showed significantly stronger inhibition effect than negative control in the group of Lfcin treatment pre-PEDV absorption, while there was no significant difference between Lfcin and negative control in the group of simultaneous addition of Lfcin with PEDV, indicating a possibility that Lfcin might affect the susceptibility of the cell receptors to PEDV, and the affinity of PEDV with cell receptors had impact on the inhibition effect of Lfcin. It has also been reported in other studies that the block of the binding between PEDV and the cellular receptors could effectively reduce PEDV infection [31–33]. Interestingly, in the group of Lfcin treatment after-PEDV absorption, whose overall inhibition effect of three different infection phases was obviously lower than that of the other two groups, Lfcin still showed a significantly stronger inhibition effect than negative control We speculated that Lfcin might activate a different cell defense mechanism after failing to block PEDV at the phase of viral entry, which needs further confirmation. As negative control, the supernatant of *Lactobacillus casei* also showed inhibitory effect on PEDV replication, indicating unspecific antiviral activity of *Lactobacillus casei* which has been also proven in other reports [34–39].

## Conclusion

In summary, we constructed a genetic engineered auxotrophy *L. casei* expressing Lfcin (*ΔthyA L.casei*), which has antibacterial and antiviral activities. This research provides a safe and effective approach for oral functional protein and other pharmaceutics purposes.

## Abbreviations

Cmr: Chloramphenicol
dTMP: Deoxythymidine uracil
dUMP: Deoxyuridine ribonucleotides
*E. coli*: *Escherichia coli*
*L. case*: *Lactobacillus casei*
LAB: Lactic acid bacteria
Lfcin: Lactoferricin
PCR: Polymerase chain reaction
PEDV: Porcine epidemic diarrhea virus
SD: Standard deviation
SDS-PAGE: Sodium dodecyl sulfate-polyacrylamide gel electrophoresis
TEM: Transmission electron microscopy
VERO: Verda Reno
*ΔthyA*: Thymidine auxotrophy
